# Comparison of Retrograde Balloon Dilatation and Laparoscopic Pyeloplasty for Treatment of Ureteropelvic Junction Obstruction: Results of a 2-Year Follow-Up

**DOI:** 10.1371/journal.pone.0152463

**Published:** 2016-03-28

**Authors:** Ning Xu, Shao-Hao Chen, Xue-Yi Xue, Qing-Shui Zheng, Yong Wei, Tao Jiang, Xiao-Dong Li, Jin-Bei Huang, Hai Cai

**Affiliations:** Department of Urology, The First Affiliated Hospital of Fujian Medical University, Fuzhou, Fujian Province, 350005, People’s Republic of China; Wright State University, UNITED STATES

## Abstract

**Objective:**

To evaluate the efficacy of laparoscopic pyeloplasty relative to retrograde balloon dilatation for the treatment of ureteropelvic junction obstruction (UPJO).

**Methods:**

This retrospective study enrolled UPJO patients with stricture length < 2 cm who had been treated with laparoscopic pyeloplasty (LP; 44 cases) or balloon dilatation (BD; 38 cases) from Jan 2010 to Jan 2012, according to patients’ preference after consultation. Demographics and clinical parameters were collected. Patients were followed-up at 3, 6, 12, and 24 months. Ultrasonography, intravenous urography, and diuretic renography were applied to evaluate the remission of hydronephrosis.

**Results:**

Both groups were comparable with respect to age, UPJO location, gender, and other baseline parameters. Compared to the LP group, patients receiving BD experienced significantly shorter operative time, analgesia time, hospital stay, and urethral catheter indwelling time, and less cost (*P*<0.001). Three and 6 months after their respective procedures, the success rates of the LP (97.7%, both) and BD (94.7% and 86.8%) groups were similar, and at 12 and 24 months the long-term success rate of LP (95.5%, both) was better than that of BD (78.9% and 71.0%).

**Conclusions:**

LP showed better long-term success rate than did BD in the management of UPJO with length of stricture < 2 cm. Considering that BD is more minimally invasive, simpler and easier to perform, and costs less, we recommend it for some selective UPJO patients as the first-line therapy.

## Introduction

Ureteropelvic junction obstruction (UPJO) is the most common cause of hydronephrosis [1]. The ideal treatment for UPJO will have a high long-term success rate, be suitable for all types of obstruction, and be minimally invasive [2]. However, such a therapy has been elusive, and open pyeloplasty remains the gold standard treatment, with success rates of more than 90%.

During the last two decades, various minimally invasive approaches have been tried for the management of UPJO, including robotic pyeloplasty, endopyelotomy, endopyeloplasty, laparoscopic pyeloplasty, and balloon dilatation. Laparoscopic pyeloplasty was initially reported by Schuessler et al. [3] in 1993, and is now considered a standard approach for the management of UPJO. Its long-term success rate is more than 90% in both adults and the pediatric population [4–6]. On the other hand, the first endourologic technique for the management of UPJO was high-pressure balloon dilatation. Although balloon dilatation is minimally invasive and simple to perform, its efficacy remains controversial, especially long-term [7–10].

The present retrospective study was conducted to compare the efficacy and safety of balloon dilatation and laparoscopic pyeloplasty in the treatment of UPJO.

## Methods

### Patients

The Ethics Committee of First Affiliated Hospital, Fujian Medical University approved this study. All patients provided written informed consent. We retrospectively reviewed the medical records of UPJO patients treated with dismembered (Anderson-Hynes) laparoscopic pyeloplasty (LP) or balloon dilatation (BD) from Jan 2010 to Jan 2012. The patients with stricture greater than 2 cm were excluded because of poor outcomes in previous reports[11, 12]. Patients without complete medical records or lost to follow-up were also excluded. At last, 44 patients who underwent LP and 38 patients treated with BD were enrolled. The following patient demographics and clinical information were collected: age, weight, gender, symptoms, UPJO location, degree of hydronephrosis, ipsilateral renal calculi, operative time, conversion to open surgery, hospitalization, analgesia time, catheter indwelling time, cost, and postoperative complications.

Before the surgery, CT angiography was routinely performed to detect crossing vessels, and ultrasonography was conducted to evaluate the degree of hydronephrosis according to the system of the Society for Fetal Urology [13]. In addition, intravenous urography/retrograde pyelography and diuretic renography using 99mTc-mercaptoacetyltriglycine were routine. The length of the obstruction of UPJ was measured by intravenous urography/retrograde pyelography. Indications for surgery were unbearable symptoms (flank pain and associated urinary tract infections), continuously increasing hydronephrosis, and impaired function of the affected kidney, based on diuretic renography findings. In each case, the type of operation was selected according to the patient’s preference after detailed explanation by the surgeon regarding the procedures, outcomes, and complications of each option. Operations were done mainly by one surgeon, Dr. Xue, who has more than 20 years’ experience performing these procedures.

### Laparoscopic pyeloplasty (LP)

General anesthesia was administrated for patients receiving LP, which in general was performed as previously described[14]. The patient was placed in the lateral position, and a 10-mm longitudinal incision was made at the tip of 12th rib along the posterior axillary line. A tunnel was created through the external oblique muscle by blunt dissection with Kocher clamp so that an index finger could push the peritoneum forward, thus creating a retroperitoneal space. A balloon made from a powder-free surgical glove and tied to the top of a ureteral catheter was placed in the retroperitoneal cavity and inflated with 500–600 mL air to produce sufficient space for the procedure. Inflation was maintained for 5 min to allow hemostasis, and then the balloon was removed. With the help of an index finger, a 10-mm trocar (for 30° laparoscope) and a 5-mm trocar were inserted 2 cm above the iliac crest and the intersection point of the costal margin and anterior axillary line, respectively. The trocar was fixed using a 0-nylon suture, to prevent carbon dioxide leakage. Then the pneumoperitoneum was established at 13–15 mmHg and a diagnostic laparoscopy was applied to check the established space. During the operation, another 5-mm trocar was inserted, if necessary.

Gerota’s fascia was incised completely and the perirenal fat dissected to reveal the lumbar ureter and the lower pole of the kidney. The UPJO was then dismembered, the ureter excised medially about 1.5 cm, and the renal pelvis was tailored appropriately. Ureteropelvic reanastomosis was performed by continuous suturing, using 4–0 polyglycolic acid sutures. A double-J ureter stent was placed in the renal pelvis before finishing the last 3 sutures. In the case of a crossing vessel, the ureter was transposed to the front of the crossing vessel. The drainage tube was removed when the volume of drainage was less than 10 mL. The Foley catheter was removed 1–2 days after removal of the drainage tube. The double-J stent was removed 6 weeks after the operation.

### Retrograde balloon dilatation (BD)

Patients were placed supine, and the operation was conducted under general anesthesia. A guide-ire crossed the stenotic segment under the guidance of an ureteroscope. A 7.5F X-Force U30 ureteroscopic balloon dilation catheter (C. R. Bard, USA) with a 4-cm balloon was introduced, and the balloon was placed across the stenotic segment. The guidewire was then removed, and an X-ray taken to outline the ureteropelvic junction and distend the renal pelvis with the help of diodone contrast. The guidewire was then re-inserted and kept until the operation was over. The balloon was inflated to a maximum diameter of 0.8 cm for 15 min. The balloon was deflated and the balloon catheter was removed after the operation. At last, a 10F double J stent was left and kept for 6 weeks. The Foley catheter was removed 1–3 days after operation. All patients were discharged after Foley catheter removal.

### Follow-up

All patients were evaluated by ultrasonography, intravenous urography, and diuretic renography at 3 and 6 months postoperatively, and semiannually thereafter. Success was defined as stability or reduction of hydronephrosis on ultrasonogram, preservation of split renal function and improvement of drainage curve on diuretic renogram, and remission of flank pain postoperatively.

### Statistical analysis

Statistical analysis was performed using SPSS, version 16.0 (SPSS, Chicago, IL, USA). Comparisons between groups were calculated by Student’s *t*-test for measurement data and Mann-Whitney U test for enumeration data. A 2-tailed *P* < 0.05 was considered statistically significant.

## Results

Between the patient groups receiving LP or BD, the baseline characteristics were similar (Table 1). S1 Table for a full list of original data. Two patients in the LP group and one patient in the BD group were diagnosed as febrile urinary tract infection and managed by culture-specific antibiotics until urine germiculture were negative.

**Table 1:** Baseline characteristics of the patients. ^a^

	LP	BD	P
Patients	44	38	—
Average age, y	30.8 (12–64)	30.5 (9–65)	0.932
Weight, kg	61.8 ± 6.4	62.0 ± 8.1	0.934
Gender, M/F	35/9	32/6	0.776
Location, left/right	27/17	23/15	1.000
Hydronephrosis ^b^			
I	2	2	—
II	9	8	—
III	15	14	—
IV	18	14	—
Presentation			0.825
Flank pain	38 (86.4%)	34 (89.5%)	—
Incidental finding	3 (6.8%)	1 (2.6%)	—
Hematuria	1 (2.3%)	1 (2.6%)	—
Lump	-	1 (2.6%)	—
Febrile UTI	2 (4.5%)	1 (2.6%)	—
Ipsilateral renal calculi	2	4	0.300
Crossing vessel	5	2	0.324
Length of UPJO, cm	1.1 ± 0.5	1.0 ± 0.5	0.419

UTI, urinary tract infection

^a^ n, unless otherwise indicated;

^b^ Society for Fetal Urology (SFU) grade

No patient required conversion to open surgery, nor blood transfusion. Two patients in the LP group and 4 patients in the BD group had concomitant ipsilateral renal calculi and were treated postoperatively with extracorporeal shock wave lithotripsy. The mean operative time of the LP group (121.60 ± 22.56 min) was significantly longer than that of the BD group (61.97 ± 8.06 min; P < 0.001; Table 2). The duration of analgesic therapy (from the completed operation to dismantling of the intravenous patient-controlled analgesia pump) of the LP group (0.71 ± 0.37 d) was also significantly longer than that of the BD group (0.23 ± 0.14 d; *P* < 0.001). The mean time of the indwelling catheter in the LP group (5.75 ± 0.60 d) was significantly longer than that of the BD group (2.43 ± 0.33 d, *P*< 0.001). LP was significantly more costly ($5552.5 ± 266.1 per patient) than BD ($2485.8 ± 167.1 per case; *P* < 0.001).

**Table 2:** Surgical outcomes of the LP and BD groups.

	LP	BD	*P*
Operative time, min	121.60 ± 22.56	61.97 ± 8.06	<0.001
Conversion	0	0	1.000
Complications	3(6.82%)	1(2.63%)	0.620
Peritoneum injury	1	—	—
Febrile urinary tract infection	1	1	—
Acute heart failure	1	—	—
Hospital stay, d	6.10 ± 0.92	4.88 ± 1.23	<0.001
Analgesia, d	0.71 ± 0.37	0.23 ± 0.14	<0.001
Urethral catheter indwelling time, d	5.75 ± 0.60	2.43 ± 0.33	<0.001
Cost, USD	5552.5 ± 266.1	2485.8 ± 167.1	<0.001
Mean follow-up, mo	36.3 (24–48)	35.5 (24–47)	0.607
Success rate			
3 mo	43/44 (97.7%)	36/38 (94.7%)	0.594
6 mo	43/44 (97.7%)	33/38 (86.8%)	0.091
12 mo	42/44 (95.5%)	30/38 (78.9%)	0.023
24 mo	42/44 (95.5%)	27/38 (71.0%)	0.005

The incidence of complications between the groups was comparable (Table 2). S1 Table for a full list of original data. Peritoneum injury occurred during dissection around the ureteropelvic junction in one patient of the LP group. The injured peritoneum was secured by Hem-o-lock clips. One patient in the LP group had acute heart failure postoperatively, and treated with a diuretic, cardiotonic drugs, vasodilators, and oxygen uptake. Febrile urinary tract infection occurred in one patient in each group; it was treated with culture-specific antibiotics.

No patient was lost to follow-up. The median follow-up in the LP and BD groups was 36 (24–48) months and 35.5 (24–47) months, respectively (*P* = 0.607). No significant difference in success rate was observed between the LP (97.7%) and BD (94.7%) groups 3 months after the procedures (*P* = 0.594), and similar results were also observed at the sixth month (97.7% and 86.8%, respectively; *P* = 0.091). LP showed better long-term success rate than BD at postoperative 12 months (95.5% cf. 78.9%, *P* = 0.023) and 24 months (95.5% cf. 71.0%, *P* = 0.005). In patients who received retrograde BD, by the end of the study those with III-IV hydronephrosis achieved only 67.9% (19/28) long-term success, while patients with I-II hydronephrosis achieved 80% (8/10).

All patients were symptom-free during the follow-up period, except for one patient in the BD group who suffered persistent flank pain and increasing expansion of the collecting system. The patient was treated with LP 5 months after retrograde BD, and then the symptoms disappeared. Ten patients in the BD group suffered persistent hydronephrosis and continuously decreasing split renal function. Seven of these patients underwent further LP, and 3 patients underwent open pyeloplasty. Two patients in the LP group received open pyeloplasty due to persistent hydronephrosis and decreasing split renal function after initial treatment.

## Discussion

We retrospectively reviewed the medical record of UPJO patients treated with LP or BD from Jan 2010 to Jan 2012, collected patients’ demographics and clinical parameters and determined the success rates of treatments at 3, 6, 12, and 24 months after the procedures. The present study indicated that short-term of success rates of LP and BD were similar, while LP showed better long-term success rates than BD. Additionally, patients receiving BD experienced significantly shorter operative time, analgesia time, hospital stay, urethral catheter indwelling time and less cost.

Previous literature reported varying success rates for BD, from 67% to 80% [8, 10, 15–17]. Sugita et al. [9] claimed, after long-term follow-up (mean 25 months), a 53% failure rate for BD performed on children. We report an overall short-term success rate for BD of 95.5%, which was comparable to that of LP. However, the long-term success rate for BD decreased to 71.0%, which was significantly lower than that of LP (95.5%).

Patients with length of stricture >2 cm are less likely to have a successful outcome after endopyelotomy [12, 18]. Ravery et al. [11] found that the mean length of obstruction in patients who had a recurrence was longer than that of patients who were cured. The patients with stricture length >2 cm had a poor outcome after long-term follow-up. In our department, all patients with stricture length >2 cm were treated with LP or open pyeloplasty, and were excluded from this study.

Sugita et al. [9] concluded that recurrent stenosis may result from excessive dilatation, which traumatizes the ureteropelvic junction and causes subsequent scarring. Intervention through BD may disrupt mucosal integrity, and the therapy finally causes localized inflammation. In addition, high-pressure or long-term dilatation can cause diffuse trauma, leading to ischemia and eventual recurrent stricture formation [19]. On the other hand, if the degree of dilatation is too slight or the dilatation time not long enough, the expansion of the UPJO will be insufficient. Parente et al. [7] believed that balloons made from poor quality materials could impair success rate in some cases; the improvement of balloons, especially increasing the pressure of low-profile balloons, would facilitate a better outcome.

Another important consideration is that BD cannot dispel pathogeny, such as cutting the crossing vessels, restoration of the kidneys. Sutherland et al. [20] claimed that UPJO in the presence of crossing vessels (which can usually be confirmed with CT angiography), should be initially treated with pyeloplasty. In the BD group, none of 4 patients with crossing vessels achieved long-term success. Osther et al. [15] found that radiotherapy-induced UPJO treated by BD had a success rate of 25%, while the success rate for congenital UPJO was 52%.

An enlarged redundant renal pelvis may also influence the outcome of BD treatment. Hydronephrosis may impair the structure and function of the ureter and renal pelvis, and therapy lessens the drainage of urine from the renal pelvis into the ureter. In the present study, this is supported by the low long-term success rate of patients in the BD group with high degrees of hydronephrosis. Sugita et al. [9] presented his opinion that a large redundant renal pelvis is prone to failure to overcome stenosis, because it causes kinking of the upper ureter. Danuser et al. [21] observed that the probability of successful endopyelotomy was higher in patients with a preoperative hydronephrosis volume < 50 mL (87%) than in patients with hydronephrosis volume > 50 mL (76%).

In our study, the mean operative time of the first 22 LP procedures was 133 min, and 110 min for the last 22, while the time for BD was much less (~62 min). It is well known that laparoscopy is much more complicated than BD and success is influenced by many factors, especially experience in suturing and knotting, and the tacit understandings among the surgical team. The learning curve for this technology is long, with some specialists suggesting that a minimum of 50 surgical procedures are needed due to its high degree of complexity, and at least one procedure per week, performed for one year, is necessary to master the essential skills [22].

Lewis-Russell et al. [8] summarized the advantages of retrograde BD as a treatment for UPJO: simple and quick, a low risk of hemorrhage, minimal morbidity, and relatively cheap. These advantages are beneficial for both individual patients and urological service providers, and make retrograde BD favorable over other procedures for first-line treatment for UPJO. Although BD could not exclude secondary endopyelotomy, laparoscopic or open pyeloplasty, it may reasonably be offered as the primary treatment in adult patients with UPJO [10, 15]. In addition, BD should be recommended to patients with poor general physical condition. In the present study, one patient in the LP group with coronary atherosclerosis had been well prepared for the procedure, but acute heart failure occurred afterward. No patient in the BD group had this kind of complication, due to the simplicity of the procedure and short operative time.

This study had some limitations. Firstly, the small study cohort and short follow-up time may reduce the reliability of the research. Secondly, given that this was a retrospective study, the evidence is not as strong as it might be in a strictly designed randomized controlled study. Randomized controlled studies with a larger cohort are needed.

In the management of UPJO, for strictures < 2 cm, LP had a better long-term success rate than did BD. Considering that BD is more minimally invasive, simpler, easy to perform, and costs less than LP, we recommend it for selected UPJO patients as the first-line therapy.

## Supporting Information

S1 Table Relevant data underlying the findings described in manuscript. (XLSX)
